# Non-Pharmacological Interventions for Managing Preoperative Anxiety in the Context of Odontectomy: A Systematic Review

**DOI:** 10.3390/dj14070428

**Published:** 2026-07-12

**Authors:** Saptiadi Oktora, Harmas Yazid Yusuf, Eriska Riyanti, Firdaus Hariri

**Affiliations:** 1 Doctoral Programme, Faculty of Dentistry, Universitas Padjadjaran, Sumedang 45363, Indonesia; 2 Department of Oral and Maxillofacial Surgery, Faculty of Dentistry, Universitas Padjadjaran, Sumedang 45363, Indonesia; 3 Department of Pediatric Dentistry, Faculty of Dentistry, Universitas Padjadjaran, Sumedang 45363, Indonesia; 4 Department of Oral and Maxillofacial Clinical Sciences, Faculty of Dentistry, Universiti Malaya, Kuala Lumpur 50603, Malaysia

**Keywords:** preoperative anxiety, odontectomy, non-pharmacological intervention, systematic review

## Abstract

Background/Objectives: Preoperative anxiety is a common issue among patients undergoing third-molar surgery, significantly impacting physiological stability, surgical outcomes, and recovery, highlighting the need for effective non-pharmacological interventions. This systematic review was performed to delineate the current literature regarding non-pharmacological interventions aimed at alleviating preoperative anxiety in patients undergoing an odontectomy. Methods: A comprehensive search was performed across major databases, including Cochrane, EMBASE, MEDLINE, PubMed, Scopus, and Web of Science, for studies published between 2011 and 2026. The inclusion criteria comprised original research, reviews, and clinical trials focused on non-pharmacological approaches to alleviating anxiety in patients undergoing an odontectomy. Results: A total of 28 studies were found via an electronic search. After screening, 11 studies were eligible for systematic review. This study demonstrates that preoperative anxiety is common in odontectomies, impacting both psychological and physiological stability. Instruments such as VAS, STAI, and MDAS demonstrate elevated anxiety levels prior to surgical procedures. This anxiety induces physiological responses that affect stability. Music therapy effectively lowers anxiety, as indicated by reduced VAS scores (F = 4.226, *p* < 0.05) and improved emotional well-being and parasympathetic activity. Conclusions: This review highlights both subjective and objective instruments for measuring anxiety as well as non-pharmacological approaches to mitigating preoperative anxiety in patients undergoing an odontectomy. While non-pharmacological interventions show potential, there is a lack of protocols and specific evidence, highlighting the need for integrated, culturally adapted strategies and regular anxiety screening.

## 1. Introduction

Oral and dental health remains a major public health concern in Indonesia [1]. According to national data from Riset Kesehatan Dasar (Riskesdas), approximately 57.6% of the Indonesian population experiences oral health problems, yet only a limited proportion accesses or receives appropriate dental care. One of the most common dental conditions is impaction of the third molars (commonly known as wisdom teeth), which often necessitates surgical intervention. If left untreated, impacted third molars may lead to serious complications, including infection, pericoronitis, and damage to adjacent teeth [2,3].

Third-molar impactions that fail to erupt properly are typically managed through a surgical procedure known as an odontectomy or surgical removal of the tooth. This is one of the most frequently performed oral surgery procedures in dental practice. However, odontectomies are associated with significant levels of preoperative anxiety, primarily triggered by anticipated pain, the use of surgical instruments, and administration of local anesthesia. A total of 45.6% of patients scheduled for third-molar extraction are categorized as anxious (31.0%) or very anxious (14.6%) based on a preoperative anxiety assessment, indicating a considerable burden of dental anxiety among patients undergoing third-molar surgery [3,4,5].

Preoperative anxiety not only affects the psychological well-being of patients but also triggers a range of physiological responses, including elevated blood pressure, an elevated heart rate, and heightened pain sensitivity. These autonomic responses may complicate intraoperative management, prolong surgical time, and delay postoperative recovery. Moreover, untreated anxiety can lead to avoidance or postponement of surgery, potentially worsening oral health conditions [6,7,8].

Pharmacological management of preoperative anxiety commonly involves anxiolytic medications such as benzodiazepines. However, prolonged use of benzodiazepines has been associated with various adverse effects, such as excessive sedation, drug interactions, a heightened risk of falls, delirium, cognitive impairment, and dependence. Due to these concerns, non-pharmacological interventions have gained attention as safer and more sustainable alternatives. These include music therapy, audiovisual stimuli, virtual reality (VR), and video. Emerging evidence suggests that these approaches can effectively reduce anxiety, improve patient comfort, and promote faster postoperative recovery [9,10].

The clinical application of non-pharmacological interventions to alleviate anxiety in patients undergoing medical procedures has demonstrated effectiveness, especially in dental environments [11]. Prior meta-analyses have demonstrated that music significantly alleviates anxiety in the context of dental procedures [12]. Nonetheless, the existing literature has not definitively established the effectiveness of music in alleviating discomfort during more invasive and painful dental procedures, including tooth extractions [13,14]. Most reviews focus on reducing anxiety in general dental procedures or concentrate exclusively on psychological interventions. An analysis specific to third-molar extraction is needed, as it is a common, anxiety-inducing oral surgery [15].

Furthermore, although there is growing clinical interest in non-pharmacological approaches, existing reviews tend to focus narrowly on specific interventions or population groups, leaving a lack of comprehensive synthesis in the context of odontectomies. To address this gap, this systematic review aims to systematically map the range of existing non-pharmacological interventions used to manage preoperative anxiety in odontectomies. Specifically, it seeks to synthesize current evidence on the types, mechanisms, and clinical outcomes of non-pharmacological interventions applied in this context. The findings of this review are expected to inform future research directions and support the development of evidence-based clinical practices in oral surgery.

## 2. Materials and Methods

### 2.1. Study Design and Framework

This comprehensive review focuses on non-pharmacological interventions carried out to reduce preoperative anxiety in patients undergoing odontectomy over a 15-year period. This review utilized the methodological framework established by Page (2021) [16]. We followed the Preferred Reporting Items for Systematic Review and Meta-Analysis extension for systematic reviews (PRISMA 2020, Table S1). The review protocol was registered in PROSPERO (CRD420261345720).

### 2.2. Literature Search Strategy

A systematic and comprehensive literature search was performed across three major electronic databases: PubMed, Cochrane Library, EBSCOhost, EMBASE, MEDLINE, Scopus and Web of Science. The search timeline spanned publications from January 2011 through January 2026, allowing incorporation of the most recent studies on the topic.

The search strategy was designed to be comprehensive, utilizing a mixture of keywords and controlled vocabulary across four pivotal domains to identify pertinent studies. The foundation search string used was as follows: “odontectomy,” “surgical tooth extraction,” and “third molar surgery”; intervention keywords such as “non-pharmacological,” “distraction,” “Cognitive Behavioral Therapy (CBT),” “music,” and “virtual reality”; outcome keywords focused on “preoperative anxiety” and “dental anxiety”; and filters for study types used to extract systematic reviews. Boolean operators (AND, OR) enabled the logical combination of these terms, with syntax tailored to fit the specific requirements of each database. To counteract publication bias, the strategy did not impose restrictions on study design, apart from the language limitations and relevancy criteria established for the search.

### 2.3. Inclusion Criteria

Studies eligible for inclusion in this review were those that satisfied several specific criteria. The population targeted consisted of patients of any age who were undergoing odontectomy or the extraction of impacted mandibular third molars. The research design had to include randomized controlled trials (RCTs), observational cohort or cross-sectional studies, or systematic reviews that specifically reported on non-pharmacological interventions. The interventions considered aimed to reduce preoperative anxiety without the use of pharmacological anxiolytic agents. Examples of accepted non-pharmacological interventions include music therapy, VR distraction, audiovisual aids, and cognitive behavioral therapy. The outcomes measured in these studies had to focus on preoperative anxiety and were assessed using validated subjective instruments such as the Visual Analog Scale, State–Trait Anxiety Inventory, and variants of the Dental Anxiety Scale. Additionally, objective physiological markers, including heart rate, blood pressure, and salivary cortisol levels, were also considered acceptable measurements for evaluating the impact of the interventions. Finally, the studies included in the review were required to be published in English and had to be available as full-text documentation.

### 2.4. Exclusion Criteria

Studies were excluded from the review based on several criteria: they could not focus solely on pharmacological management of anxiety or combine pharmacological and non-pharmacological interventions without a distinct analysis. Additionally, studies related to dental procedures that were not associated with odontectomy or that were non-surgical were excluded. Furthermore, the exclusion criteria prohibited conference abstracts, editorials, commentaries, or narrative reviews lacking original data, as well as studies published in any language other than English.

### 2.5. Study Selection Process

**Figure 1:**
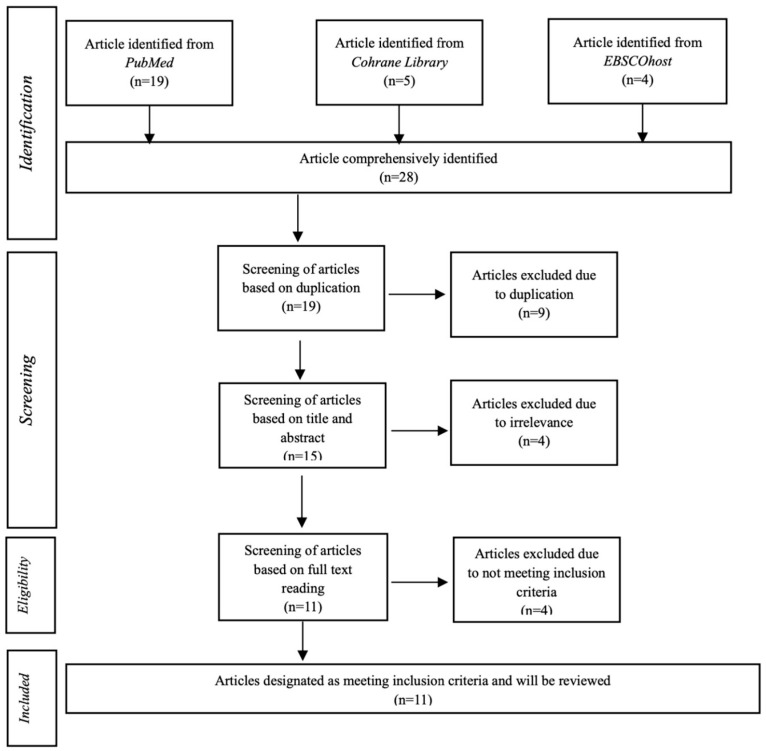
Diagram of the research item identification and section process.

The selection of studies proceeded in a systematic manner and was divided into two distinct phases. The first phase involved title and abstract screening, where two independent reviewers evaluated all titles and abstracts based on pre-defined inclusion and exclusion criteria to identify studies that could be relevant. Any discrepancies arising from this assessment were addressed through discussion or by consulting a third reviewer to reach consensus. The second phase was the full-text review, during which the same reviewers retrieved and independently assessed the full texts of studies deemed eligible for final inclusion. Any disagreements encountered at this stage were similarly resolved through consensus discussions. Additionally, the entire study selection process was documented using a PRISMA flow diagram to ensure transparency and adherence to guidelines (Figure 1).

### 2.6. Data Extraction

A standardized data extraction form was developed and piloted on a selection of articles. The extracted data included study characteristics (authors, publication year, country, and study design), participant demographics (sample size, age range, and significant clinical or demographic features), intervention details (type, duration, method of delivery, and timing in relation to surgery), anxiety measurement tools used (both subjective and objective), primary and secondary outcomes related to anxiety reduction efficacy, and key findings, alongside reported limitations of each study. Data extraction was performed independently by two reviewers to ensure accuracy and completeness, with discrepancies resolved via discussion.

### 2.7. Study Quality Assessment

To assess the quality of the studies, we utilized the revised Cochrane risk-of-bias tool for randomized trials (RoB 2), focusing on five domains: randomization process risk, intervention deviation bias, missing-outcome-data bias, outcome measurement bias, and reported result selection bias. Two independent reviewers conducted the assessment and reached consensus through discussion.

### 2.8. Data Analysis and Synthesis

Extracted data were synthesized thematically. Studies were grouped according to the type of non-pharmacological intervention under investigation (e.g., music therapy, audiovisual distraction, or VR). For each category, findings regarding efficacy, mechanisms of anxiety reduction, and outcome measurement were summarized descriptively. Both subjective measures (psychometric scales) and objective physiological markers were analyzed to foster a comprehensive understanding of intervention effects. Narrative synthesis was chosen given the heterogeneity in interventions, outcomes, and study designs, precluding meta-analytic pooling. Gaps and inconsistencies in the literature were critically appraised to identify priorities for future research and potential clinical translation.

## 3. Results

### 3.1. Study Selection and Characteristics

A total of 11 studies met the predefined inclusion criteria and were included in this systematic review. The majority of these studies were randomized controlled trials (RCTs, *n* = 8), while the remaining three studies were observational in design. Sample sizes varied widely, ranging from as few as 40 participants to as many as 225, reflecting differences in study scope and resource availability. Participants across studies were predominantly adults between 18 and 60 years old undergoing odontectomy procedures, with no studies exclusively focusing on pediatric or geriatric populations. The geographic distribution of the studies included countries with varying levels of healthcare infrastructure, although many originated from medium- to high-income settings. Table 1 provides a concise summary of the studies that were included in the review.

### 3.2. Types of Non-Pharmacological Interventions

The included studies collectively evaluated several types of non-pharmacological interventions aimed at reducing preoperative anxiety associated with undergoing an odontectomy. The most frequently examined modality was music therapy, investigated in six studies, with interventions ranging from passive listening to playing specific genres or frequencies of music during the surgical procedure or immediately beforehand.

Audiovisual aids, including informative procedural videos or audiovisual treatment information, were assessed in three studies. These interventions typically involved patient education or distraction techniques that visually and auditorily engaged patients prior to or during surgery.

VR technology, an immersive distraction tool providing simulated environments unrelated to the surgical experience, was evaluated in two observational studies and one randomized clinical trial. VR was used mainly intraoperatively to divert patient attention and alleviate anxiety during local anesthesia administration. An additional modality explored was the use of binaural-beat music, which targets brainwave entrainment to induce relaxation, as studied in one observational trial.

### 3.3. Anxiety Measurement Instruments

A variety of subjective and objective tools have been utilized in studies to assess levels of anxiety, particularly within dental contexts. Among the subjective measurements, the Visual Analog Scale (VAS) for anxiety stands out as a continuous scale that permits patients to quantify the intensity of their perceived anxiety. Another prominent tool is the State–Trait Anxiety Inventory (STAI), which effectively distinguishes between transient state anxiety and the more persistent trait anxiety. Additionally, several dental-specific anxiety scales have been validated for assessing anxiety related to dental procedures, including Corah’s Dental Anxiety Scale (CDAS) and the Modified Dental Anxiety Scale (MDAS). Each of these tools plays a crucial role in accurately gauging anxiety levels in patients undergoing dental treatment.

To enhance the reliability of self-reported data, numerous studies have incorporated objective physiological markers that indicate autonomic arousal and stress responses. These markers include measurements of both systolic and diastolic blood pressure, monitoring of heart rate (also referred to as pulse rate), tracking of respiratory rate, and analysis of salivary cortisol levels. Salivary cortisol serves as a critical biomarker reflecting the activation of the hypothalamic–pituitary-adrenal (HPA) axis during instances of stress, providing an objective quantification of stress-related physiological changes. The simultaneous use of subjective and objective measures in some studies enhanced reliability and provided multidimensional insight into anxiety modulation.

### 3.4. Effectiveness of Interventions

Music therapy: Most studies reported significant reductions in subjective anxiety scores among patients exposed to music therapy compared to controls. For example, the randomized controlled trials by Kim et al. (2011) [17] and Maulina et al. (2017) [19] showed statistically significant decreases in VAS (F = 4.226, *p* < 0.05) and DAS scores, ranging from 7.3 to 8.7, during both the preoperative and intraoperative periods. Beyond psychological effects, music therapy also demonstrated beneficial modulation of heart rate and blood pressure, indicative of sympathetic nervous system attenuation. Variations in music genre and frequency (e.g., 432 Hz vs. 440 Hz) yielded mixed but generally positive results, suggesting that personalized music selection might optimize anxiety reduction [23]. Furthermore, a study conducted by Kupeli & Gülnahar (2020) [21] revealed that preoperative and intraoperative classical music significantly reduced anxiety in patients aged 18–30 years undergoing third-molar extraction compared to other music genres.

Audiovisual aids: Studies utilizing educational and procedural videos reported anxiety alleviation primarily through enhanced patient understanding and perceived control over the surgical process. Choi et al. (2015) [18] demonstrated a significant decrease in STAI and hemodynamic parameters, supporting the hypothesis that reducing uncertainty lowers anxiety. Toledano-Serrabona et al. (2020) [24] similarly found that an informative video played before mandibular third-molar extraction significantly improved anxiety scores and stabilized hemodynamics compared to standard care. Furthermore, dental anxiety, prevalent in adults, was reduced using audiovisual aids during tooth extraction, showing significant postoperative score improvements compared to verbal instructions in a study of 162 participants [26].

Virtual reality: VR interventions offered immersive distraction with promising preliminary outcomes. Yamashita et al. (2019) [22] reported reduced anxiety levels and lower heart rates during extraction procedures under local anesthesia. However, the findings were limited by small sample sizes and heterogeneity in VR content and duration. Mladenovic & Djordjevic (2021) [25] reported that VR is a feasible tool for reducing both pain and anxiety, though barriers such as cost and technology accessibility were acknowledged. VR use during dental treatment significantly reduced anxiety among patients, with objective measurements supporting improved autonomic balance and no cybersickness, as reported by Yamashita et al. (2020) [20].

Binaural-beat music: Salehabadi et al. (2024) [27] found binaural-beat music effective in lowering dental anxiety scores and salivary cortisol levels, suggesting potential neurophysiological benefits. Nevertheless, evidence remains limited, and further large-scale randomized studies are warranted.

### 3.5. Additional Observations

Several studies noted that anxiety reduction was not uniform across all patient subgroups [28,29,30]. Factors such as baseline anxiety, previous dental experiences, and individual preferences appeared to modulate intervention effectiveness [31,32,33]. Few studies addressed long-term impacts such as reductions in dental avoidance behavior or postoperative recovery trajectories [34]. Moreover, few studies focused on culturally adapted interventions or populations with medically compromised statuses.

## 4. Discussion

Preoperative anxiety during an odontectomy is a prevalent and clinically significant issue that affects both psychological well-being and physiological stability. This review confirms the high prevalence of anxiety prior to third-molar surgery, as measured by validated instruments including VAS, STAI, MDAS, and CDAS. Anxiety in this context extends beyond emotional distress and is closely associated with autonomic nervous system activation and hypothalamic–pituitary–adrenal (HPA) axis stimulation, resulting in increased heart rates, elevated blood pressure, and heightened cortisol secretion [34]. These physiological responses may compromise intraoperative hemodynamic stability, increase analgesic requirements, and prolong postoperative recovery, underscoring the critical need for effective anxiety management strategies [35,36,37].

Music therapy significantly influences brain function, particularly in emotional processing and regulation [38]. It modulates amygdala activity, which is central to fear and anxiety, as evidenced by neuroimaging studies (fMRI and PET) showing decreased hyperactivation linked to negative emotions. This reduction alleviates anxiety and depression symptoms. Concurrently, music therapy activates the prefrontal cortex (PFC), enhancing emotional regulation and cognitive control. Improved connectivity between the PFC and amygdala following music therapy indicates better emotional integration. The combination of decreased amygdala reactivity and enhanced PFC regulation creates an adaptive neural network, offering a targeted, non-invasive alternative to pharmacological treatments for emotional health.

Various non-pharmacological interventions have demonstrated potential in mitigating preoperative anxiety. Music therapy, the most frequently investigated approach, is thought to activate the parasympathetic nervous system and modulate emotional responses through limbic structures, thereby alleviating anxiety symptoms [39]. Audiovisual aids, such as procedural videos, reduce uncertainty and enhance patients’ perceived control, both of which are key mediators in anxiety reduction. VR offers immersive distraction by diverting attention from the surgical environment, which may be particularly beneficial for patients undergoing procedures under local anesthesia [40]. Nevertheless, despite promising preliminary findings, VR interventions currently lack standardized protocols concerning optimal duration, content, and timing. Furthermore, challenges related to technological access and cost may limit the feasibility of VR implementation in low-resource settings [18,20,22].

Outcome measures across studies exhibit considerable heterogeneity. While subjective anxiety scales are widely utilized due to their ease of administration, they remain susceptible to response bias and individual differences in self-reporting. Objective physiological markers, including salivary cortisol, heart rate, and blood pressure, provide more quantifiable and reliable data but require specialized equipment and trained personnel [41]. Studies incorporating both subjective and objective assessments tend to yield more robust findings, emphasizing the need for methodological standardization in future research to enhance comparability and reliability [42,43].

However, significant gaps remain in the current literature. Most studies have been conducted in controlled clinical settings involving healthy adult populations, which limits the generalizability of findings. Evidence is scarce regarding pediatric, geriatric, and medically compromised patients as well as the long-term impact of interventions on dental avoidance or phobia. In addition, feasibility challenges in resource-constrained environments may hinder the widespread adoption of VR and other technologically dependent interventions. This study highlights the necessity of integrating non-pharmacological strategies into future clinical guidelines, providing specific recommendations for their implementation across diverse patient populations and oral surgery contexts. This approach would standardize and optimize preoperative anxiety management in common odontectomies and impact psychological well-being and physiological stability, thereby improving patient outcomes and satisfaction [44,45].

Future research should emphasize inclusive sampling, culturally adapted approaches, and standardized protocols to enhance the clinical applicability of non-pharmacological anxiety management in oral surgery. Additionally, it should prioritize well-designed randomized trials focusing on music-based interventions during third-molar surgery, establishing uniform protocols for music selection, exposure duration, and outcome measures for consistent study comparisons. The NHS England Clinical Standards for Dental Anxiety Management published in 2023 listed patient anxiety and cooperation, dental treatment urgency and invasiveness, and existing comorbidities as key factors in the model of care for special needs patients [46]. Non-pharmacological management, including light sedation with agents like nitrous oxide, is safe in primary care settings. Moreover, incorporating patient-selected music and tailored auditory approaches may improve therapeutic outcomes. Evidence supports the notion that music therapy effectively manages anxiety and enhances patient experiences during surgery.

In this systematic review, the authors acknowledge that there are still limitations, including the following: (a) providing quality assessment in the methodology; (b) misunderstanding the performance of meta-analysis; (c) publication bias; (d) limited geographic diversity; and (e) short follow-up periods that prevent assessment of long-term effectiveness. Additionally, the heterogeneity in music type; duration; study timing, particularly regarding music protocols; outcome measures; and study design may limit the comparability of the results.

## 5. Conclusions

This systematic review has discussed various strategies for managing dental anxiety in odontectomy patients in the dental setting. Implementing a combination of the techniques discussed is recommended for dentally anxious patients. Music therapy significantly reduces subjective anxiety scores among patients, with randomized controlled trials showing decreases in VAS and DAS scores during preoperative and intraoperative periods. It also positively modulates heart rate and blood pressure. Selection of personalized music, particularly classical, can enhance anxiety reduction. Audiovisual aids improve patient understanding and perceived control, leading to lower anxiety scores and stabilized hemodynamics. Virtual reality provides immersive distraction, reducing anxiety and heart rates during dental procedures, but it faces limitations like small sample sizes. Binaural-beat music shows potential in lowering anxiety and salivary cortisol, although further large-scale studies are needed to draw conclusive evidence.

While non-pharmacological interventions show promising outcomes, the lack of standardized protocols and population-specific evidence limits their widespread implementation. Future studies should focus extensively on the potential of these technologies to reduce dental anxiety and enhance behavior management in typical odontectomy settings. This approach will help dentists and caregivers ensure proper treatment for this vulnerable population.

## Figures and Tables

**Table 1:** Summary of included studies.

**Author**	**Year**	**Title**	**Research Design**	**Sample Size**	**Anxiety** **Assessment Tools**	**Intervention** **Duration**	**Outcomes/Conclusions**
Yu-Kyoung et al. [17]	2011	Musical intervention reduces patients’ anxiety in surgical extraction of an impacted mandibular third molar	Randomized controlled trial	219 patients	Musical intervention	October 2008 to June 2009	The use of patient-selected music during IMTM surgical extraction significantly reduced the patient’s intraoperative anxiety levels.
Sung-Hwan et al. [18]	2015	Effect of audiovisual treatment information on relieving anxiety in patients undergoing impacted mandibular third-molar removal	Randomized clinical trial	51 patients	Audiovisual treatment	December 2014 to March 2015	Audiovisual presentation significantly enhances patient knowledge and reduces anxiety.
Tantry et al. [19]	2017	The effect of music intervention on dental anxiety during dental extraction procedure	Randomized clinical trial	225 patients	Music intervention	March to November 2016	Islamic music reduces dental anxiety to a significantly greater extent than classical music.
Yoshio et al. [20]	2019	Clinical effect of virtual reality to relieve anxiety during impacted mandibular third-molar extraction under local anesthesia	Randomized clinical trial	51 patients	Virtual reality intervention	April to November 2018	VR significantly enhances dental treatment, especially during surgery.
Ilke et al. [21]	2019	Comparing different music genres in decreasing dental anxiety in adolescents who underwent third-molar tooth surgery in turkey—randomized controlled trial	Randomized controlled trial	80 patients	Music genre intervention	June to November 2019	Classical music during surgery significantly reduced anxiety in young patients.
Kaoru et al. [22]	2019	The effects of music listening during extraction of the impacted mandibular third molar on the autonomic nervous system and psychological state	Observational	40 patients	Music-listening intervention	March to September 2018	Music during molar extraction reduces sympathetic nerve activity and anxiety.
Pedro et al. [23]	2019	Effect of music at 432 Hz and 440 Hz on dental anxiety and salivary cortisol levels in patients undergoing tooth extraction a randomized clinical trial	Randomized clinical trial	40 patients	Music intervention	June to September 2018	Music significantly reduced clinical anxiety and salivary cortisol before tooth extraction.
Jorge et al. [24]	2020	Effect of an informative video upon anxiety and hemodynamic parameters in patients requiring mandibular third-molar extraction: a randomized clinical trial	Randomized clinical trial	50 patients	Informative video intervention	January 2018 to November 2019	Video information significantly reduces patient anxiety and heart rate preoperatively.
Rasa et al. [25]	2021	Effectiveness of virtual reality as a distraction on anxiety and pain during impacted mandibular third-molar surgery under local anesthesia	Observational	74 patients	Virtual reality (VR) intervention	February to November 2020	VR distraction during third-molar surgery significantly reduces anxiety and pain.
Ijaz et al. [26]	2023	Role of audiovisual aid in reduction of dental anxiety during tooth extraction: a randomized clinical trial	Randomized clinical trial	162 patients	Audiovisual aid intervention	September to December 2022	Audiovisual aids reduce dental anxiety during tooth extractions significantly.
Negareh et al. [27]	2024	Can Binaural beat music be useful as a method to reduce dental patients’ anxiety?	Observational	80 patients	Binaural-beat music intervention	10 September to 19 October 2022	Binaural beats significantly reduce anxiety non-pharmacologically in patients.

## Data Availability

No new data were created or analyzed in this study. Data sharing is not applicable to this article.

## Supplementary Materials

The following supporting information can be downloaded at: https://www.mdpi.com/article/10.3390/dj14070428/s1, Table S1: PRISMA 2020 checklist.
